# The Caregiver Pathway, a Model for the Systematic and Individualized Follow-up of Family Caregivers at Intensive Care Units: Development Study

**DOI:** 10.2196/46299

**Published:** 2023-04-25

**Authors:** Solbjørg Watland, Lise Solberg Nes, Elizabeth Hanson, Mirjam Ekstedt, Una Stenberg, Elin Børøsund

**Affiliations:** 1 Department of Digital Health Research Division of Medicine Oslo University Hospital Oslo Norway; 2 Medicine Intensive Care Unit, Department of Acute Medicine Division of Medicine Oslo University Hospital Oslo Norway; 3 Institute of Clinical Medicine Faculty of Medicine University of Oslo Oslo Norway; 4 Department of Psychiatry and Psychology College of Medicine and Science Mayo Clinic Rochester, MN United States; 5 Department of Health and Caring Sciences Linnaeus University Kalmar Sweden; 6 Swedish Family Care Competence Centre Kalmar Sweden; 7 Department of Learning Informatics Management and Ethics Karolinska Institutet Stockholm Sweden; 8 Norwegian National Advisory Unit on Learning and Mastery in Health Oslo University Hospital Oslo Norway; 9 Frambu Resource Center for Rare Disorders Ski Norway; 10 Department of Nursing and Health Sciences Faculty of Health and Social Sciences University of South-Eastern Norway Drammen Norway

**Keywords:** caregivers, next of kin, relatives, family-centered care, digital assessment tool, nurse intervention, intensive care unit, ICU, post–intensive care syndrome-Family, PICS-F, empower, support, follow-up, health promotion

## Abstract

**Background:**

Family caregivers of patients who are critically ill have a high prevalence of short- and long-term symptoms, such as fatigue, anxiety, depression, symptoms of posttraumatic stress, and complicated grief. These adverse consequences following a loved one’s admission to an intensive care unit (ICU) are also known as post–intensive care syndrome-family. Approaches such as family-centered care provide recommendations for improving the care of patients and families, but models for family caregiver follow-up are often lacking.

**Objective:**

This study aims to develop a model for structuring and individualizing the follow-up of family caregivers of patients who are critically ill, starting from the patients’ ICU admission to after their discharge or death.

**Methods:**

The model was developed through a participatory co-design approach using a 2-phased iterative process. First, the *preparation phase* included a meeting with stakeholders (n=4) for organizational anchoring and planning, a literature search, and interviews with former family caregivers (n=8). In the subsequent *development phase,* the model was iteratively created through workshops with stakeholders (n=10) and user testing with former family caregivers (n=4) and experienced ICU nurses (n=11).

**Results:**

The interviews revealed how *being present* with the patient and receiving *adequate information* and *emotional care* were highly important for family caregivers at an ICU. The literature search underlined the overwhelming and uncertain situation for the family caregivers and identified recommendations for follow-up. On the basis of these recommendations and findings from the interviews, workshops, and user testing, *The Caregiver Pathway* model was developed, encompassing 4 steps: within the first few days of the patient’s ICU stay, the family caregivers will be offered to complete a digital assessment tool mapping their needs and challenges, followed by a conversation with an ICU nurse; when the patient leaves the ICU, a card containing information and support will be handed out to the family caregivers; shortly after the ICU stay, family caregivers will be offered a discharge conversation by phone, focusing on how they are doing and whether they have any questions or concerns; and within 3 months after the ICU stay, an individual follow-up conversation will be offered. Family caregivers will be invited to talk about memories from the ICU and reflect upon the ICU stay, and they will also be able to talk about their current situation and receive information about relevant support.

**Conclusions:**

This study illustrates how existing evidence and stakeholder input can be combined to create a model for family caregiver follow-up at an ICU. *The Caregiver Pathway* can help ICU nurses improve family caregiver follow-up and aid in promoting family-centered care, potentially also being transferrable to other types of family caregiver follow-up.

## Introduction

### Background

Family caregivers, hereafter referred to as caregivers, of patients admitted to intensive care units (ICUs) frequently experience the situation as traumatic. The patients’ distress often impacts the whole family [1], and caregivers are worried about the patient. The admission to the ICU could be dramatic, and the shock and uncertainty render the caregivers vulnerable [2,3]. Models for caregiver follow-up in these dire situations have been called for [1,4].

Fatigue, anxiety, depression, complicated grief, and posttraumatic stress symptoms are among the long-term consequences experienced by caregivers of patients who are critically ill [4-7]. This cluster of consequences is also known as post–intensive care syndrome-family (PICS-F), the adverse complications among caregivers after the ICU stay of patients with critically illness [5]. There is a high prevalence of PICS-F, and symptoms of complicated grief have, for example, been found in over half of the caregivers who lost their loved ones owing to such critical illness [8].

The many psychosocial consequences of an ICU admittance require new approaches, and over the recent years, family-centered care [9] has increasingly gained attention in hospitals, as focus has shifted from centering on providers to centering on patients and their families, including on how to best support and meet their needs [10]. The family-centered care approach involves being respectful and responsive and acknowledging the situation and needs of individual patients and their families [9].

Acute stressors and the feeling of being overwhelmed or overburdened among caregivers have been found to predict PICS-F [11]. Therefore, assessing caregivers’ distress and offering caregiver support interventions at an early time point [1], as well as helping them make sense of what has happened or is happening and helping them understand their new role as caregivers [5], have been recommended. Continued attention and support throughout, from the patient’s admission to after their discharge or death, have also been highlighted as important [12]. Caregivers who have lost their loved ones because of critical illness need new approaches for facilitating healthy grieving processes. If their loved one has recovered, is still recovering, or may never completely recover, more continuous follow-up or support may be needed.

Caregivers often serve as a liaison between the patient and health care provider and between the patient and their social network [13]. Their health literacy, described as their personal health-related knowledge and ability to access, understand, and use services and information to maintain their own and their loved ones’ well-being and health [14], is, therefore, important for both the caregivers themselves and the patients. Caregivers’ health literacy varies and often also depends on health care organizations’ proficiency to provide services supporting caregivers’ ability to seek and receive adequate health support [15]. ICU nurses are in close contact with patients and their families and are vital in the follow-up of caregivers, identifying factors and symptoms contributing to psychological distress and subsequently being able to offer support at an early stage [1]. With ICU nurses already having challenging and compiled workloads, it is increasingly evident that they also need support and resources to further improve the quality of the care they deliver [16]. Timely support may enhance the caregivers’ health literacy, give them the support they need, and hence potentially prevent long-term consequences such as symptoms of posttraumatic stress, fatigue, anxiety, and depression.

Follow-up and support for caregivers of patients in the ICU are clearly needed. Recommendations for improving the follow-up of caregivers exist [9]; however, models for individualizing and structuring such support are generally lacking, and novel, implementable models designed for caregiver support at the ICU are called for [1,4,11,12].

### Aim

This study aimed to develop a model for structuring and individualizing the follow-up of caregivers of patients who are critically ill, starting from the patients’ admission to the ICU to after their discharge or death.

## Methods

### Design

The study adopted a participatory design approach in which users (ie, former caregivers and experienced ICU nurses) were actively involved and acted as co-designers [17,18]. The planned model for caregiver follow-up was expected to consist of several interactive components and was, therefore, classified as a complex intervention model [19]. The Medical Research Council’s guidelines for the development and evaluation of complex interventions were hence used during the development process [19,20]. The Medical Research Council’s *guidance on how to develop complex interventions to improve health and health care* recommends using a combination of existing theories and frameworks and expert opinions or experiences and describes actions needed during development to produce successful health interventions [20]. The model was named *The Caregiver Pathway* and subsequently anchored and developed based on stakeholder input and the identified effective recommendations for caregiver follow-up.

**Figure 1:**
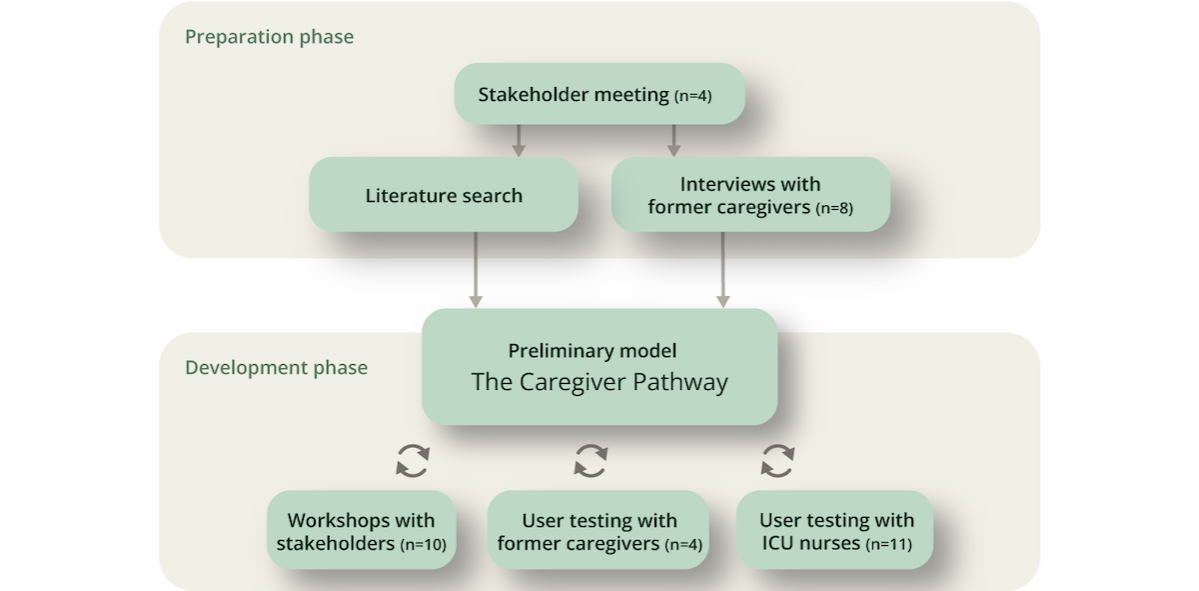
The preparation and development phases of *The Caregiver Pathway* model. ICU: intensive care unit.

The model development was supported by user input gathered through an iterative process, starting with a *preparation phase* consisting of a stakeholder (ie, managers and experienced ICU nurses at a Medical ICU; n=4) meeting for organizational anchoring and planning; a literature search for exploring caregiver symptoms, challenges, and needs and identifying recommended interventions for supporting caregivers; and interviews with former caregivers (n=8). On the basis of the findings from the preparation phase, a draft of the preliminary model was outlined at the beginning of the *development phase* to elicit stakeholder and user input. The *development phase* consisted of workshops with stakeholders (ie, managers and experienced ICU nurses; n=10) to discuss usability and feasibility and refine the preliminary model, followed by user testing with former caregivers (n=4) and ICU nurses (n=11). Refer to Figure 1 for an illustration of the model preparation and development processes.

### Setting

*The Caregiver Pathway* model was developed in collaboration with the Medical ICU and the Department of Digital Health Research located in the Division of Medicine at Oslo University Hospital, Norway.

### Data Collection and Analysis

#### Preparation Phase

##### Stakeholder Meeting

A stakeholder meeting was initially held with managers (n=2) and experienced ICU nurses (n=2) in charge of the educational training program at the Medical ICU to ensure organizational anchoring and project support and identify potential concerns and reflections related to the support and follow-up of caregivers at the unit. The first (SW) and last (EB) authors took notes during the meeting, and meeting minutes were sent to the stakeholders, with comments and additional input encouraged.

##### Literature Search

The literature search for the model development was conducted on PubMed in March 2019 using the following search terms: “intensive care unit,” “ICU,” “critically ill patient,” “trauma,” “caregivers,” “family,” “next of kin,” “follow-up,” and “support.” The inclusion criteria were as follows: articles with adult caregivers of patients in ICUs written in English or a Scandinavian language. In addition, governmental web pages were searched, and national guidelines were identified. The initial PubMed search generated 82 scientific articles. The abstracts were screened, and articles concerning parents of newborns and children or patients only were excluded, as caregivers of newborns and children have roles different from those of caregivers of adults. Articles not relevant to the ICU setting were also excluded, for example, articles whose main focus was on palliative care at home. The remaining 52% (43/82) of articles were read in full text, and 16 (37%) of these contributed specifically to the model development through concrete recommendations for caregiver follow-up.

##### Interviews With Former Caregivers

Using purposive sampling, experienced ICU nurses at the Medical ICU asked former caregivers who had been at the ICU (median 6 months ago; range 3-8 months ago), either by phone or during a follow-up conversation, whether they were interested in participating in individual interviews that would help provide information for the development of a caregiver support model. The ICU nurses strived to include caregivers and bereaved former caregivers, males and females, with a variety of story types to tell and diverse experiences and relationships with the patients (eg, parents, spouses, and children) to strengthen diversity among informants. All former caregivers who were contacted (n=8) agreed to participate. The 8 caregivers had a median age of 49 (range 38-65) years, 3 were male (38%; ie, n=2, 67% spouses and n=1, 33% parent), and 5 were female (62%; ie, n=4, 80% spouses and n=1, 20% daughter). Of the 8 participants, 3 (38%) were bereaved caregivers of patients who passed away while in the Medical ICU. The interviews were conducted by the first author, who is also an ICU registered nurse, using a semistructured interview guide (Multimedia Appendix 1). Participants were asked to describe the support received at the ICU, as well as their needs and preferences as a caregiver during their stay at the ICU, after discharge, and following the death of their loved one. They were encouraged to reflect upon what was important to them and what might have been lacking for them in the time past their ICU experience. In addition, participants were asked about their current situation, whether they received any support during or after the stay at the ICU, and whether they had any thoughts or suggestions for caregiver follow-up.

The interviews were transcribed verbatim by the first author and uploaded to the software program NVivo (version 12, QSR International). The transcripts were then read through by the first and last authors, and a thematic analysis approach was adopted. The thematic analysis started with a descriptive analysis [21], focusing on what was experienced as supportive or challenging by the caregivers. The content was coded into predefined categories related to the patient timeline: (1) admission, (2) being at the ICU, (3) discharge, (4) home again, and (5) mourning. Subcategories describing the challenges and indicative areas for improvement were then identified and guided the development of the preliminary model.

The thematic analysis continued with further examination of the subcategories and transcribed text, and information underlining how to improve the support of caregivers were identified and abstracted into subthemes and thereafter main themes.

#### Development Phase

##### Workshops With Stakeholders

A preliminary draft of the model was outlined, and various parts of the model were discussed in relation to usefulness and feasibility with the stakeholders (ie, managers and experienced ICU nurses at the Medical ICU, n=10). The stakeholders were invited to participate in 5 workshops over a 14-month period. The last author moderated the workshops and ensured that all participants were allowed to express their opinions. A summary of the recommended interventions for supporting caregivers identified through the literature search and findings from the caregiver interviews were presented during the workshops. The first and last authors took notes during the workshops, summarized them in meeting minutes, and distributed them to participants after the workshops, encouraging comments and additional input. The preliminary model was refined until there was consensus within the stakeholder group to further develop the model via user testing.

##### User Testing

Of the 8 former caregivers who participated in the interviews, 4 (50%; ie, n=2, 50% female and n=2, 50% male, bereaved, as well as current caregivers; age range 38-65) were asked by the first author whether they were interested in contributing as user participants in the subsequent development phase. All of them agreed to participate. Experienced ICU nurses at the Medical ICU were asked by the manager of the Medical ICU to also help test the model, and 11 agreed, 3 (27%) of whom were already involved in the project through the workshops.

Former caregivers (n=4) and experienced ICU nurses (n=11) provided feedback and further input on the preliminary model (Figure 1). Potential content for a digital assessment tool for mapping caregivers’ needs and challenges was first presented as a paper draft, and participants were asked to provide input on the content and wording in several iterations. The co-designed content was then implemented in a digital solution and iteratively tested using a tablet, followed by input-based adjustments. In addition, the text to be used as information and support for caregivers and guidelines for conversations with caregivers after the hospital stay were approved. Former caregivers received gift cards of approximately €25 (US $25) to cover travel expenses when contributing user input. The ICU nurses contributed to the project on a voluntary basis during their working hours.

### Ethics Approval

The study was approved by the hospital institutional review board equivalent (ie, Personvernombudet at Oslo University Hospital; 19/09470). All procedures were conducted in accordance with existing ethical standards [22]. Participating former caregivers signed an informed consent form and were informed that they could withdraw from the study at any time without any adverse consequences to any existing treatment or support they might be receiving. Personal identification was removed from the transcripts of the individual interviews and replaced with a study-specific code. No personal details that could be potentially linked to individual participants were stored in the meeting minutes. If the former caregivers experienced any form of distress during the study, they were encouraged to contact the first author at any time, who would guide them toward the appropriate resources for support.

## Results

### Preparation Phase

#### Stakeholder Meeting

Stakeholders (ie, managers and experienced ICU nurses at the Medical ICU) described not only several positive experiences due to the ability to support caregivers through conversations and practical help but also how it could sometimes be challenging to provide caregivers the support need. In particular, the stakeholders described the limited time and resources available to support caregivers and highlighted a need for the new model to fit into the hectic ICU environment, stating that caregiver follow-up should also be a good experience for the ICU nurses. As long as these factors were taken into consideration, there was a consensus to develop a model for improving caregiver follow-up.

The stakeholder meeting also identified a need for a literature search and interviews with former caregivers. An adjusted version of an existing digital assessment tool, previously found useful for assessing patients’ needs and preferences [23], was suggested as a means to facilitate the assessment of caregivers’ needs and preferences. A stakeholder meeting agreement was made to adjust and implement the updated digital assessment tool in the model.

#### Literature Search

The conducted literature search identified existing approaches to caregiver support and follow-up, as well as symptoms, challenges, and needs among caregivers. The search underlined the needs for an individual and a structural caregiver follow-up and identified the following recommendations for supporting caregivers: a *“get to know” conversation* [12]; an *information leaflet about the ICU* [9]; the identification of susceptible caregivers by *screening for anxiety and depression* [7,24]; the *identification of the feeling of being overburdened* [11]; an *assigned nurse* to assure continuity [25]; *flexible visiting hours* for facilitating *caregivers’ involvement in patient care* [9]; a *discharge conversation* [12]; a follow*-*up *conversation after discharge* [12,26]; and a *diary for the patient*, which potentially also improves the situation for the caregivers [27,28]. A model for supporting the unique needs of caregivers was described as lacking [4], and strategies for reducing the stressful experiences of caregivers were identified as called for [11,12]. Refer to Multimedia Appendix 2 [2-4,9,11,12,24,25,28-35] for an overview of how the literature contributed to and inspired the development of the final model. The governmental caregiver guideline describing caregivers’ rights, health care services’ obligation to offer caregiver follow-up, and recommendations for good practice inspired and was fundamental in supporting the model development [36].

The literature search further revealed how caregivers described their experiences in the ICU as overwhelming. They reported feeling unprepared for the situation and the uncertainty and described having to wait for information and not knowing what the outcome would be as very difficult [2,3,11]. Feelings of helplessness, with emotions appearing to alternate among sadness, worries, fear, uncertainty, hope, and despair [3], as well as a feeling of being overburdened [11], were described; these feelings and emotions could ultimately place caregivers in a vulnerable situation [2].

The literature search showed how the ICU nurses were considered to be in a unique position to understand and support caregivers [4]. The caregivers’ confidence in the care received by the patient [2] and the respect and dignity shown by the health care personnel to the patient were identified as being of great importance [3]. In addition, adequate, timely, and consistent information [3,29]; continuity in care; and the ability to communicate with the same provider [25] were also found to be much appreciated. The caregivers were seen as wanting to be present with the patient and to protect their loved one in the traumatic situation, and through the support from the ICU nurses, some also wanted to be engaged in the active care of the patient [2,3,29]. Being at home was described as difficult, with their loved one’s critical illness affecting their whole life, and many reported not having the strength to manage daily tasks, such as doing chores in the house or homework with their kids [3]. The caregivers described constantly waiting for the telephone to ring, with difficulties getting enough sleep [3]. The caregiver room at the hospital, where caregivers could rest and recuperate, was reported to be important, and other family members were described as a source of support, as was the opportunity to talk with other caregivers in similar situations [3].

Findings from the literature search also identified the caregivers’ situation as changing throughout the period from the patient’s ICU admission to after their discharge, with caregivers reporting being relieved and hopeful in the beginning and thereafter feeling uncertain [37]. As many patients do not recover to prehospital functioning after discharge, some caregivers were found to feel overwhelmed by consequent responsibility [12,37]. The caregivers described having to stay vigilant and having to modify routines at home, as their loved one could not do the same activities as before, with caregivers having to set new goals and realistic expectations for them [37]. The literature search showed how the caregivers themselves were unlikely to get enough rest, not having time to exercise or recuperate and forgetting to take care of their own health and medical needs [30]. The search also showed how the critical illness had a long-term effect on caregivers, with decreased health-related quality of life and symptoms of anxiety, depression, and posttraumatic stress being commonly reported [4-7].

Despite all the identified challenges, the literature search also revealed how some caregivers experienced meaning through the difficulties, describing feeling strong and not having considered themselves capable of going through such a difficult time [3]. The literature search showed that hope was found to be of great importance, even if the prognosis was negative [3,29], potentially reducing symptoms of posttraumatic stress [6].

#### Interviews With Former Caregivers

##### Overview

The interviews with former caregivers confirmed several findings identified from the literature search, such as the need for information, caregivers being in a vulnerable situation, and caregivers feeling afraid and overwhelmed, underlining the need for the improvement of caregiver follow-up during and after the ICU stay. In the first phase of the thematic analysis, data were examined and allocated into groups according to the patient stay at the hospital and then into subcategories describing areas for the improvement of follow-up during and after the stay at the ICU. The subcategories from this descriptive analysis were explored and incorporated into *The Caregiver Pathway* model. Refer to Table 1 for quotes from the caregiver interviews and details related the subcategories and how these were addressed in *The Caregiver Pathway* model.

**Table 1:** Examples of caregiver interview quotes, subcategories, and model impact.

Timeline and quotes			Subcategories		Addressed in the model
**Being at ICU^a^**					
	“It has been an awful long time, I was here all the time, I saw how it developed, and thought oh no, I have to be here, because when the day comes, I am walking here alone...That’s why I tried to be there as much as possible” (C3^b^).	Importance of being with the patient		Question included in the assessment tool	
	“My mum wanted the window to be open, she preferred to have it cold. Many of the nurses said, ‘I have put an extra jacket on today, so we can have the window open.’ I thought ‘you should not sit and be cold,’ but at the same time it was nice, they really tried everything” (C8).	Trusting the health care providers		Question included in the assessment tool	
	“For me it was hard to be at home, when I was home, I was not peaceful, I took pills because I was so tired. In 1 or 2 months, I lost 10 kilograms” (C7). “Some said we should remember to eat when we came home, and it was good they reminded us” (C8).	Enough rest and nutrition		Question included in the assessment tool	
	“I am sure we were very demanding as caregivers, especially my oldest daughter needed to control everything” (C1).	Need for information		Question included in the assessment tool	
	“Thinking about it afterwards, everything was just moving along, what I really would have needed was someone taking me aside” (C6).	Need for individual support		Individual conversation with the nurse	
**Discharge**					
	“I could not rejoice when he was getting better, I was afraid if he was to move to another unit it would be the end for him. The physician told me he was going to be discharged, I told them, no, he cannot come home, I cannot look after him...He changed so quickly, he was discharged and after two days he was readmitted” (C7).	Transition to ward was challenging		Topic included in discharge conversation	
**Home again**					
	“I am exhausted, it is hard to be with someone who is depressed and anxious” (C1). “I am exhausted, I have held myself up for the sake of my mum and the boys” (C2).	Being exhausted		Related text included in the supportive card	
	“If you are a patient and do not demand anything, or just accept what you are given, you are not being prioritized, so I have been a demanding caregiver, and all the way made sure we got what we should have” (C4). “I need to get help, I need to talk with my general practitioner about it. I have not had time to do it, but now, I must take the time needed” (C7).	The importance of being proactive		Related text included in the supportive card and topic included in the follow-up conversation	
	“I have reduced my working hours; he cannot be on his own for long” (C1). “I have been on sick leave, and I have been back at work for a period, right now, I am on 40% sick leave...It is a proactive thing from my side, I recognized I needed a timeout so I would not simply crash” (C4).	Need for reduced workload or facilitation from workplace		Related text included in the supportive card and topic included in the follow-up conversation	
**Mourning**					
	“My wife sat down with me and got me to see myself as she did. Then I scheduled an appointment with a psychologist...I feel there is such a pressure...I hope it will help, I go around and I am very tense” (C5). “I have asked a lot of times, how do I go on with life? What do people do? OK, I have to go on alone...There is no recipe, you have to make it yourself. Are there no places you can contact for help and guidance?” (C3).	Dramatic experiences and need for processing and guidance		Related text included in the supportive card and topic included in the follow-up conversation	
	“I remember I told one of the nurses that this has to be over, I cannot do it anymore. I cannot believe I said so, but I could not cope with it any longer” (C8).	Feeling guilty		Topic included in the follow-up conversation	

^a^ICU: intensive care unit.

^b^C: caregiver.

Following the examination, the descriptive subcategories and transcribed text were sorted into subthemes of importance for improving caregiver follow-up. The examination of the subthemes revealed interconnections, and 3 main themes emerged as particularly important for caregiver follow-up: *being present*, *receiving adequate information*, and *emotional care*.

##### Being Present

The importance of being with their loved one was highlighted by the caregivers, who wanted to be with the patient as much as possible and believed that nothing could substitute this. Being with the patient was found to give comfort in the critical situation, and the caregivers also reported being worried that the patient might feel left alone if they were not present:

I would not leave him. I was there all the time, it felt better to be here...At the worst I thought he was going to die, I would not leave him alone.

C7

The caregivers reported that being present made them feel that they were doing something, even in a situation where there was not much that they could do for their loved one. They described how the uncertainty of the situation made them restless when at home, always waiting for the phone to ring, and also described the atmosphere at the ICU as comforting, as the nurses provided a space for them and often gave them a *break* by talking about day-to-day things. One of the caregivers expressed the following in this regard:

I got to be with her when she was in a coma, and the nurses let me ask questions and talked with me about everyday things, it was good.

C4

When transferred to another unit or hospital, the desire to be with the patient remained, although with a shift in focus. The caregivers expressed the need to look after the patient and ensure that appropriate help was provided. Many described how the patient did not want to be alone after returning home. Some described needing flexibility in work or the ability to reduce working hours to be with the patient as required.

##### Receiving Adequate Information

The uncertainty surrounding the situation with the loved one was described by many as burdensome. The fear of losing the patient or the patient remaining in a severe condition was reported as particularly challenging. Receiving adequate information was described as “something to hold on to” in the uncertainty. The information reportedly helped the caregivers understand what had happened or was going to happen with their loved one. Adequate information, described as timely, clear, and consistent information, appeared to ease the uncertainty and comfort the caregivers and was reported to be important for trusting the medical staff. Concrete information was reported to facilitate the handling of other issues arising in the acute situation. The caregivers also described daily meetings with physicians offering information as being appreciated, with these meetings acting as an opportunity for them to be involved. Some caregivers also reported choosing to describe their own concerns in these meetings, such as their worries about the patient’s outcome:

We had daily meetings, I asked, “what do you think?” a lot...I was very “on,” because I needed to know, I wanted to be involved. I said so many times; the goal is to save him, and bring him home, but not regardless of the prize, he needs to be saved to a life worth living.

C3

The caregivers described not getting enough information or that the information was unclear and stated that this caused additional stress. The transition to other units was also described as challenging because of the shift in information routines. For example, information from the physicians was provided to the patients while they were recovering, but the patients did not always manage to share the information with the caregivers. Therefore, the caregivers reported sometimes suddenly being in a new state of uncertainty without access to information. They described information as crucial for them to feel prepared, for example, for challenges in the recovery phase, such as when experiencing unexpected reactions from the patient:

When he was confused, he could not do anything. One time he did not recognize me either, he was not my husband any longer. We were not prepared, we did not get the information that it could happen, that he could be confused, or that it would be exhausting for me as well.

C7

Caregivers stated that they were informed that they would have a lot to process and would benefit from seeking support or that they would have to initiate seeking such support themselves.

##### Emotional Care

The caregivers described being a caregiver to a patient who is critically ill as highly challenging. They reported being very worried and having to wait a lot and described a feeling of powerlessness, as there was little that they could do for the patient. In these fragile situations, respect and caring words from the ICU nurses were considered to be of help, with caregivers assigning great value to the health care personnel observing their needs and seeking to alleviate the situation for them. Practical care was also reported to make the caregivers feel emotionally supported, such as offering a glass of water or physical space in a gentle manner and encouraging and facilitating rest. The caregivers described being comfortable talking about ordinary things and difficult feelings such as anger and sadness with the ICU nurses. They reported valuing the competence of and support communicated by the ICU nurses, including the nurses’ ability to provide hope:

As caregivers, we were very well-taken care of. Humanity, ethical competence, the ability to reflect, are important when you meet patient and caregivers, and you had all of it. You gave us hope with a realism, you did not give us unrealistic expectations, but a combination of support and understanding.

C1

Emotional care was highlighted as vital even after the hospital stay, as the caregivers described still having a lot to deal with and process. Challenges experienced at the ICU stuck with them. They also expressed having been exhausted to the extent that they could not cope with the situation any longer and wanted it all to end. In other words, they sometimes wanted their loved one to pass away so that they could get out of the situation, something that they reported feeling guilty about afterward. They also reported feeling guilty for not bringing the patient to the hospital fast enough and questioned whether earlier treatment could have prevented the critical illness.

The importance of social support and returning to work was also described, with caregivers stating that the workplace could act as a social arena. They also reported wanting the patient to return to work, as this could alleviate the situation for the caregivers as well:

...his goal is to get his drivers’ license back and get back to work. I hope it will happen before the winter. He should go out more, and he should let me go out more. I think he will cheer up if he goes out more, positive input from others would help.

C2

The feeling of being isolated when returning home was frequently expressed by the caregivers, describing how their work and social life had changed and that the need for emotional support was evident. The caregivers also stated that when they were ready, they had to start their social life over again, and some reported being the ones making the first move:

I think it is easier for people that I have been open when they have met me. People are clumsy, ridiculously clumsy, and as I say to people, it is much better to be straight forward, do not try to avoid the issue. Then you just steal energy from the one who is troubled. Just say you think it is difficult. I understand it well, don’t beat around the bush. What has happened has happened. It is much better to be straightforward.

C5

The caregivers reported having a lot to vent about and process after the critical incident with their loved one, regardless of whether their loved one had survived or passed away. The amount of support that the caregivers reported receiving varied considerably. Some reported having received professional help, whereas others said that they had not received any kind of support, although they recognized that they needed it.

### Development Phase

#### Workshops With Stakeholders

Recommendations identified through the literature search, combined with the findings from the interviews with former caregivers, were presented in stakeholder workshops and formed the basis of the preliminary model of *The Caregiver Pathway*. The preliminary model was then discussed in terms of its usefulness and feasibility in the Medical ICU. The digital assessment tool meant to assess the caregivers’ situation, followed by a conversation with an ICU nurse, was regarded as a feasible means to structure and individualize support for the caregivers. Topics identified as important to address during the ICU stay were discussed, and a consensus was reached as to whether these should be included in the digital assessment tool. To ensure that information and support were provided to all caregivers, creating a supportive card to be delivered with information at patient discharge was also suggested. Identified challenges related to transitions to other units and returning home were also discussed, and the inclusion of a discharge conversation was suggested for the model. A follow-up conversation after patient discharge was also recognized as key, and topics from the interviews were included in the follow-up conversation script.

**Figure 2:**
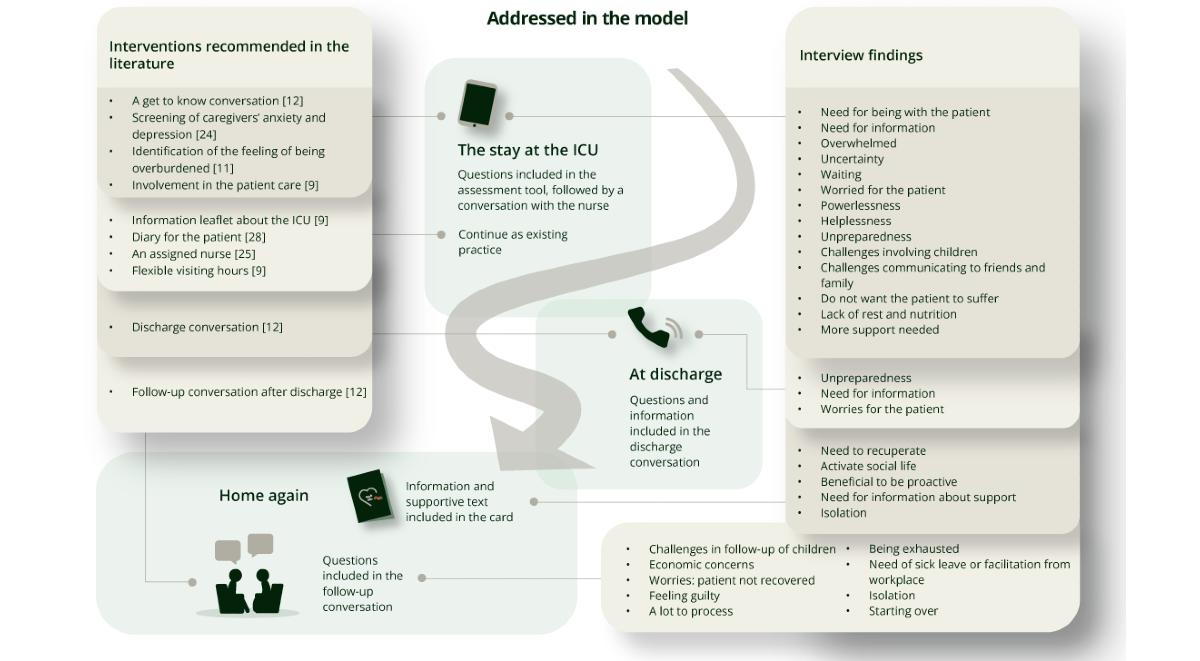
*The Caregiver Pathway* model content. ICU: intensive care unit.

Refer to Figure 2 for an overview of how the recommendations identified in existing literature and findings from the caregiver interviews were discussed in the stakeholder workshops and eventually addressed in the model.

#### User Testing

All inputs from the user testing by former caregivers and ICU nurses were considered, explored, deliberated, and executed until a consensus was reached. Specifically, the content and wording in the digital assessment tool and supportive card underwent multiple adjustments based on feedback from user testing. The aim was for the model to be suitable for a wide range of caregivers, regardless of gender, age, the disease that caused the ICU admission, or whether the critical illness led to death and bereavement. With this in mind, the preliminary model underwent several iterations, user testing, and revisions for optimization. The feasibility for nurses to use *The Caregiver Pathway* model and the need for the model to be easy to use, practical, and easily accessible, thus inducing motivation for use among the ICU nurses, were emphasized.

### Finalizing the Model

#### Overview

**Figure 3:**
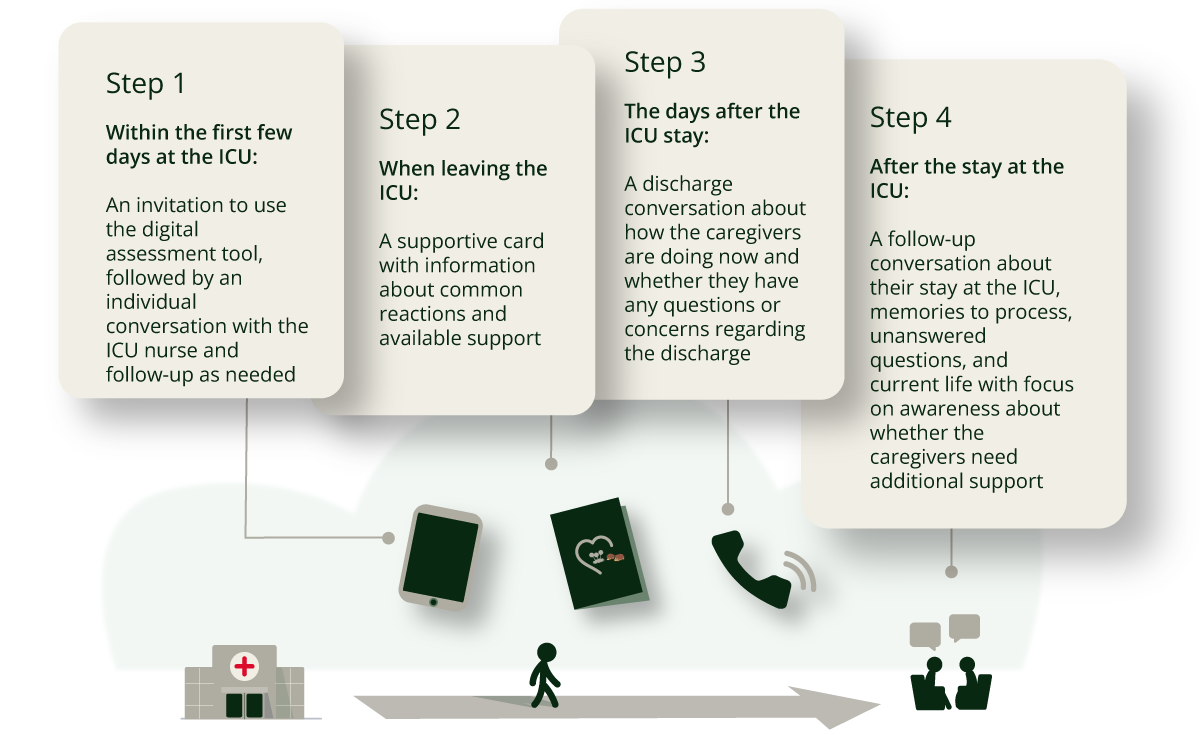
*The Caregiver Pathway* model. ICU: intensive care unit.

After several iterations, the model was approved by the former caregivers and experienced ICU nurses in the study. The final version of *The Caregiver Pathway* model consists of four steps: (1) caregivers will be asked to complete a digital assessment followed by a conversation with an ICU nurse at the beginning of the patient’s ICU stay; (2) at discharge, caregivers will receive a card with information and a supportive text; (3) in the first 1 to 2 days after discharge, caregivers will have the opportunity to take part in a follow-up conversation by phone; and (4) within 3 months post-ICU stay, caregivers (ie, bereaved or not) will be offered an individual follow-up conversation. Refer to Figure 3 for an illustration of *The Caregiver Pathway* Model.

#### Step 1: The Digital Assessment Tool Followed by a Conversation With an ICU Nurse

**Figure 4:**
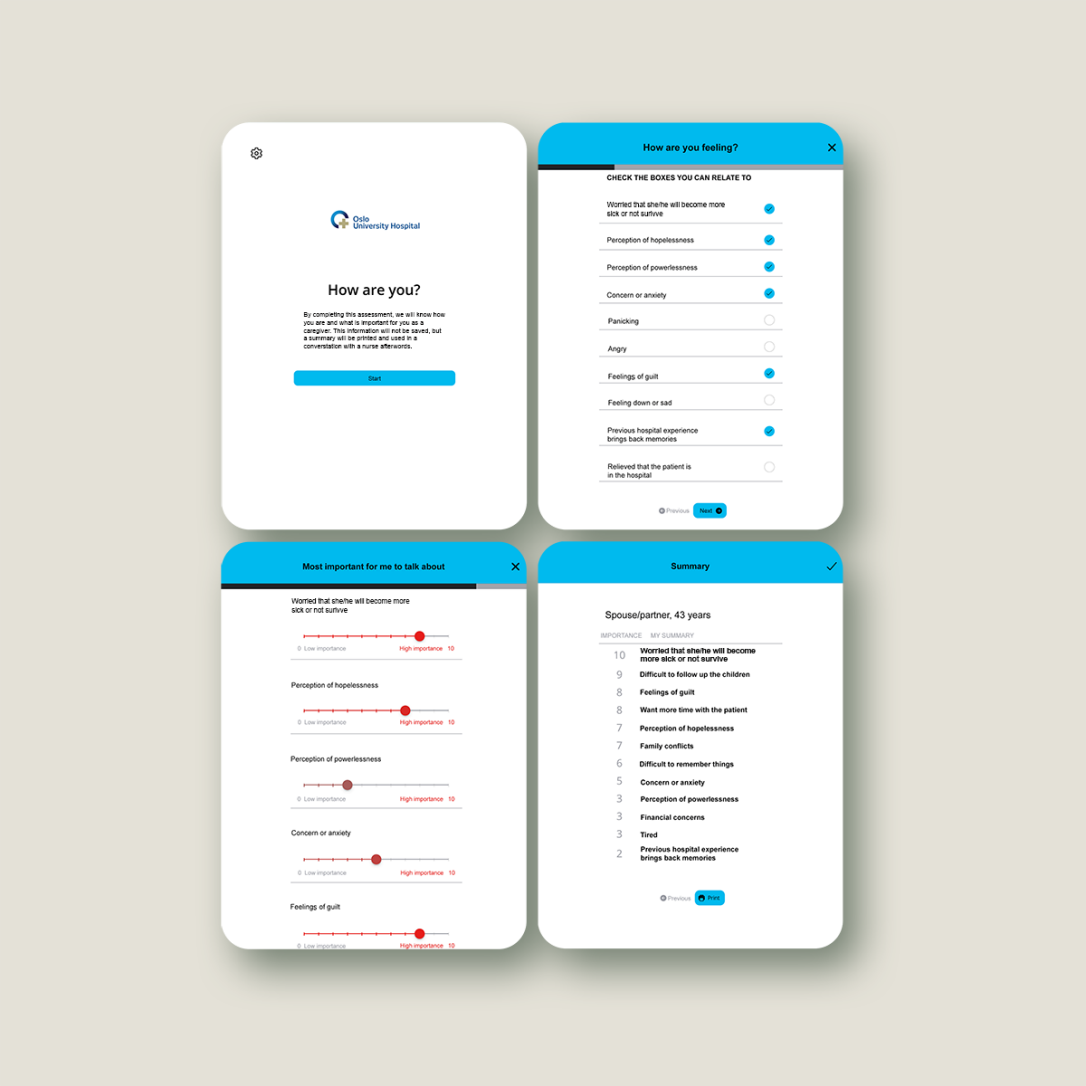
The digital assessment tool—screenshot examples.

The digital assessment tool aims to help caregivers articulate their needs and preferences in a structured manner. Refer to Figure 4 for screenshot examples of this tool. The assessment tool is designed to be administered through a tablet, and the first page provides caregivers with an introduction to the assessment. Next, they can mark their needs and preferences as caregivers and thereafter grade their answers according to what they need to talk about the most. Finally, the assessment tool generates a summary of what the caregiver would like to prioritize discussing. The summary can be printed and is to be subsequently used in a conversation with an ICU nurse, who can identify the individual’s needs for help and ensure that such help is provided (eg, referring the caregivers to an appointment with a hospital social worker).

#### Step 2: The Supportive Card

**Figure 5:**
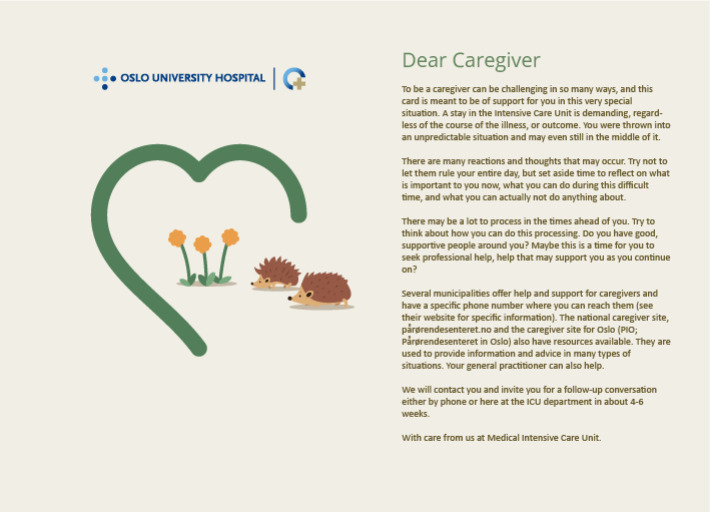
The supportive card.

The supportive card contains information acknowledging the challenging situation that the caregiver has experienced or is still experiencing. The information is intended to help caregivers become aware of common reactions and to encourage them to seek help if needed. In addition, the card provides information about the available support and is an invitation to the follow-up conversation (Figure 5).

#### Step 3: The Discharge Conversation

The discharge conversation is to be offered in the form of a phone call, 1 to 2 days after the patient has been discharged from the ICU. The care and information routines often vary depending on the medical unit, particularly when the patient is no longer critically ill. Regardless of the situation after discharge, caregivers may have several concerns related to the condition that the patient was or is in, and the need for information may be substantial. In this discharge conversation, caregivers will be asked about *how they are doing now and whether they have any questions or concerns.* Some concerns are common for caregivers, and an immediate conversation with an experienced ICU nurse can be of great value. If the patient has moved to another unit with the same degree of follow-up, this discharge conversation may not be needed, as other nurses may look after them. In addition, if a patient has passed away, a phone call may not be appropriate at such an early point in the grieving process. Therefore, the discharge conversation should be offered only if it is considered appropriate.

#### Step 4: A Follow-up Conversation

Caregivers will be invited to a follow-up conversation within 3 months after the patient’s discharge or death. Caregivers can choose whether to meet at the hospital or have the conversation over the phone. The follow-up conversation will be semistructured (ie, will follow a template), and an ICU nurse who knows the caregivers from the ICU stay will conduct the conversation, encouraging them to talk about their memories from the ICU. Caregivers will be asked whether *there was anything that they found helpful, anything that they found difficult, or anything that they did not understand or wanted to be different.* The aim is to clarify unanswered questions and help caregivers eventually process difficult situations, providing them with the opportunity to reflect upon the ICU stay. If considered beneficial or necessary, the physician who was involved in the treatment of the patient will also be invited to participate.

The conversation will end with a focus on the person’s present life, and the ICU nurse will pay particular attention to whether the caregiver, or now a former caregiver or bereaved person, is in need of additional support, and if so, the nurse will help facilitate such support by informing the caregiver about centers and organizations available for caregivers.

## Discussion

### Principal Findings

#### Overview

This study involved the development of *The Caregiver Pathway*, a structural model that aims to improve the follow-up of caregivers of patients who are critically ill. The findings from a stakeholder meeting at the participating ICU, in combination with a literature search in the area, confirmed the need for the improvement of caregiver follow-up, identifying caregivers’ experiences of unpreparedness, uncertainty, being overwhelmed, and being in a vulnerable situation as the main topics of importance. Interviews with former caregivers identified 3 main themes as vital for the caregivers: *being present* with the patient, *receiving adequate information*, and *emotional care.* Thereafter, a preliminary model was developed, discussed in stakeholder workshops, and iteratively user tested; thus *The Caregiver Pathway* model was finalized*.*

#### Being Present

Being present with the patient was one of the main themes identified in the caregiver interviews, but the extent to which caregivers want to be present and involved in the care of the patient may vary. Existing research supports letting the family be present and actively involved during patient care [2,9,38] and encourages ICU nurses to create a culture of partnership with caregivers [10,38]. This is also supported by the theory of nurse-promoted engagement with families in the ICU, which identifies the facilitators and disrupters of ICU nurses’ engagement with caregivers, proposes strategies, and underlines the importance of families being a part of the patients’ care [39]. Involvement of the family is deemed to prevent the development of PICS-F [9], as emphasized in family-centered care [9]. However, family involvement is not necessarily straightforward to implement in practice, and there is still a discrepancy between how much caregivers want to be present and involved in the care of the patient and how much they actually are involved [40].

The procedures involved in taking care of the patient, the ICU environment, and lack of time have been described as obstacles to caregiver involvement in patient care [41]. The ICU nurses have to balance the patients’ needs for integrity, the caregivers’ needs for involvement, and their own work situation and obligations [41], and collaboration with and support of caregivers often come in addition to other complicated and challenging tasks for these nurses [16]. Supporting caregivers can be stressful for the ICU nurses who, at the same time, have to perform complicated patient treatment, and flexible ICU visiting hours can increase the risk of burnout among ICU nurses [42]. This has contributed to voices of warning about implementing flexible visiting hours at the cost of ICU nurses [43], despite caregivers’ wishes to be being present.

Considering the situation of caregivers as well as that of the ICU nurses, *The Caregiver Pathway* model aims to facilitate communication with caregivers, pointing to ways of assessing and supporting caregivers’ level of involvement, with the ICU nurses carrying the main responsibility of facilitating caregiver involvement [4,7,31,39,40,44]. The questions included in the digital assessment tool (step 1) in *The Caregiver Pathway* are guided by these aspects, and by using the tool, caregivers can get an opportunity to express their needs and wishes, with the tool potentially facilitating a dialogue about what the caregivers prefer and what the ICU unit can offer them. *The Caregiver Pathway* can thus facilitate involvement and help promote more family-centered care.

#### Adequate Information

Receiving adequate information was another main theme identified through the caregiver interviews in this study, and information about their loved one’s condition could be described as an anchor for the caregivers in the uncertainty. Daily conversations with the physicians usually cover information about the patient’s status, and these conversations are often followed up by bedside discussions with the ICU nurses. However, information about how to take care of themselves and their own health and well-being is also crucial for caregivers, and in line with the findings from other studies [1,11,12,37], the findings from this study show how caregivers did not experience being offered information and advice concerning their situation as a caregiver, pointing to a need for structured models to address the issue.

Information and advice about the caregiver’s own situation is an essential part of *The Caregiver Pathway*. The digital assessment tool (step 1) enquires about caregivers’ needs for information and explicitly asks whether they need various kinds of support. The subsequent conversation and initiatives from the ICU nurses also aim to ensure that adequate help is provided and that information about how to handle the new situation as caregivers or bereaved caregivers is provided. The supportive card (step 2) offers information about common reactions and directs caregivers to relevant support. In *The Caregiver Pathway* model, the caregivers are also offered a discharge conversation (step 3) to meet their information needs, for example, due to transition to other units or home, and a follow-up conversation (step 4) aimed at helping caregivers process their memories from the ICU and at detecting and clarifying potential misunderstandings in the hectic hospital environment. Providing clarification can help caregivers process challenging experiences. *The Caregiver Pathway* follow-up conversation also centers on the caregiver’s situation at the moment, and the ICU nurses must pay attention to whether appropriate help is received and, if necessary, provide information and encourage the caregivers to seek help, for example, through supportive caregiver centers and organizations.

*The Caregiver Pathway* model seeks to also target caregivers who do not have the strength, skills, or education needed to maneuver the health care system. In other words, the model aims to strengthen health literacy by providing health information required to reach out for health-related social support and to strengthen the ability to engage with health care personnel and navigate the health care system [45]. Situational difficulties can negatively impact any caregiver’s health literacy, and a structural individual follow-up, such as that indicated in *The Caregiver Pathway* model, can contribute to information and support reaching those who need it the most. The model also shows how the assigned ICU nurse could offer additional phone calls after discharge if needed, and the follow-up of caregivers by the hospital or their general practitioner has been recommended for at least 6 months after an ICU stay [24], with the main responsibility for further follow-up being assigned to the caregiver’s general practitioner.

#### Emotional Care

Emotional care is essential for caregivers of patients who are critically ill, as caregivers may have a wide range of emotional reactions and need to be given room to be comfortable expressing them [13]. The caregiver interviews in this study identified emotional care, anything from facilitating rest to saying the right words or, more importantly, taking the time to listen, as the third main theme of importance for caregiver follow-up. The caregivers also valued the ICU nurses giving them hope yet at the same time being realistic and not giving them false hope and expectations.

The importance of articulating feelings is at the base of *The Caregiver Pathway* digital assessment tool (step 1), which contains questions related to common emotions with the intention of helping caregivers recognize and accept feelings that they may not know are usual in such situations, ultimately contributing to identifying and reducing distress. The assessment tool also asks whether the caregivers would like to have a conversation with a priest, counselor, or social worker, as individuals from these professions can also contribute to helping the caregivers through this rough period in life.

There is a large variation in how ICU nurses practice emotional care and caregiver follow-up [38,44]. The way in which they meet the caregivers could be a result of traditions at the ICU, combined with available resources for caregiver follow-up [39,41], and improved general communication strategies with caregivers are needed [11]. The caregivers in this study described the conversations with the ICU nurses to be of irreplaceable value, and it should be noted that spontaneous conversations could be as meaningful as structural meetings [2]. However, offering structural communication, such as that indicated in *The Caregiver Pathway* model, can ensure that all caregivers are offered a consultation where important topics can be covered, and ICU nurses as such have a unique opportunity to detect and prevent negative consequences that might occur among caregivers of patients who are critically ill [4,7,31,39,40,44]. They may also provide a glimpse of hope, which has been described as being of great importance [3,29], potentially preventing PICS-F [6].

#### The Development Process

The participatory design used in this study allowed former caregivers, ICU managers, and experienced ICU nurses to act as co-designers, thereby facilitating a democratic environment with subsequent user testing and adjustments. The evidence- and theory-based initiations of *The Caregiver Pathway* model were hence supported by the deep insights gained from the user contributions. Such a person-based approach to development highlights the perspective of the potential end users who are going to actually use the intervention [46] and is intended to enrich and complement the traditionally theory-based approach [46], supporting the model in reaching its purpose. The user-centered approach used is also beneficial for gaining deeper insights and reveal the psychological, social, ergonomic, and organizational factors affecting a product [17]. In addition, the engagement of users, as seen in this study, may increase the likelihood of the intervention’s implementation in clinical practice, as could iterative input and user testing, which help tailor the intervention to suit contextual needs [20]. The user-centered development of *The Caregiver Pathway* also took flexibility and adaptation to individual caregivers and contexts into account, additional success factors for the implementation of interventions in health care [47].

#### Before Implementation

When implementing complex interventions, it is essential to be aware of their potential adverse effects [48]. The ICU nurses may experience emotional tensions and frustration when taking care of caregivers, specifically owing to a lack of resources and facilities [44], and it is important to avoid putting more demands on the ICU nurses than what they have the resources for. Having to take care of caregivers without organizational support can be a lonely and frustrating experience [44], and *The Caregiver Pathway* should not be implemented without the support and resources from the ICU management. However, given the availability of such support and resources, caring for caregivers can be rewarding, despite the many challenging experiences of working in the ICU.

Another potential adverse effect is that implementing the model may give caregivers expectations that may be difficult to fulfill, which makes the follow-up of needs or concerns raised by caregivers in such a model crucial. Caregiver follow-up also requires several instances of working together, and collaborations within the hospital as well as with the broader health care system are needed to relieve the burden of the caregivers and ICU nurses providing such care [12,44]. It is also important to be aware of existing resources, such as centers and organizations providing valuable support to family caregivers. Helping caregivers become aware of such resources is an essential component of the proposed model. Tailoring *The Caregiver Pathway* to suit the local context is also a vital factor for its acceptance and successful eHealth implementation [49].

In the Medical ICU where *The Caregiver Pathway* model was developed, they already practiced approaches in support of caregivers, including providing information leaflets upon admission, an assigned nurse, flexible visiting hours, and a diary for the patient. Research shows that it can be beneficial for some caregivers to keep a diary during the stay at the ICU [50], and a recent study showed that diaries written by caregivers with assistance from ICU nurses had the potential to reduce the level of symptoms of posttraumatic stress among caregivers [51]. Reading and talking about their experiences with their loved one might also be helpful for the caregivers and patients. In ICUs not already offering such support, information leaflets, an assigned nurse, flexible visiting hours, and the use of a diary could, therefore, potentially be included for beneficial caregiver follow-up.

### Limitations

This study has several limitations. First, the first author worked at the included Medical ICU as an ICU nurse and knew the managers and experienced ICU nurses participating in the study workshops. This could have possibly impacted participants’ willingness to honestly express their opinions. However, all participants expressed a wish to contribute to improved caregiver follow-up and willingly shared their experiences and ideas. It should also be noted that the last author had no relation to any of the participants and moderated the workshops. Knowing the context could also be regarded as an advantage, as this and being familiar with the challenges faced by patients, caregivers, and ICU nurses can be crucial for the development of an intervention or a model [52].

Second, and along similar lines, the fact that all participants were in the same ICU environment could limit the intervention’s representativeness and potential generalizability to other contexts.

Third, the first author, who conducted the interviews with former caregivers, knew the caregivers from the ICU stay. This could have impacted the details and the way in which they were shared by the participating caregivers in the interviews. However, the caregivers were contacted by other ICU nurses for participation and had already agreed to participate before the involvement of the first author. In addition, several participants described feeling comfortable talking to a person who knew what they had gone through.

Fourth, the first authors’ prior understanding of the context and knowledge of the participants could have potentially influenced the analysis of the interviews. However, the analysis was thoroughly discussed with the last author, who was naive to the context and participants, to increase the trustworthiness of the analysis before reaching a consensus on interpretation.

Finally, the original digital assessment tool was designed to elicit the patients’ symptoms, needs, and preferences. Therefore, whether the adjusted tool truly captures the spectrum of caregivers’ needs remains to be seen. However, the version developed in this study was based on user input and adapted to fit the setting experienced by caregivers with loved ones in the ICU, including topics important to such caregivers. *The Caregiver Pathway* digital assessment tool was also iteratively tested by caregivers and ICU nurses to ensure suitable content for caregivers.

### Conclusions

This study illustrates how existing evidence and stakeholder and user input can be combined to co-design and create a model for caregiver follow-up at an ICU. The variety of methods used to design *The Caregiver Pathway* outlined essential elements such as being present, receiving adequate information, and emotional care for the support of caregivers at critical time points during patient care and follow-up. The final *Caregiver Pathway* model can, through its 4-step structural and individual perspective, improve support for all caregivers and be a useful tool for the ICU nurses in their everyday work with caregivers of patients who are critically ill. Future studies should test the model in a randomized controlled trial to evaluate its feasibility and impact, and if effective, the model may be implemented and potentially also transferred to other ICUs and areas of the health care system.

## Appendix

Multimedia Appendix 1 Manual for interviews with former caregivers.

Multimedia Appendix 2 An overview of how the literature contributed to and inspired the development of the final model.

## Abbreviations

ICU: intensive care unit
PICS-F: post–intensive care syndrome-family

## Data Availability

The data sets generated in this study are not publicly available because of current national and organizational privacy and security legislations in protection of participants’ confidentiality.
