# Supplementary material for: The Caregiver Pathway, a Model for the Systematic and Individualized Follow-up of Family Caregivers at Intensive Care Units: Development Study
Source: JMIR Form Res. 2023 Apr 25;7:e46299. doi: 10.2196/46299 (PMC10170368; doi:10.2196/46299)
Supplement: Multimedia Appendix 1 [file formative_v7i1e46299_app1.pdf]

Manual for interviews with former caregivers

The Caregiver Pathway

Introductory question: How are you doing now?

Is there anything in particular we could have done different when you were a caregiver at our ICU unit?

Were there any needs you were not able to inform us about?

Did you feel like the Health care personnel had time for you?

Did we do anything that you appreciated?

Was there anything that you did not appreciate?

Our goal is to take care of everyone, do you have any thoughts about how other caregivers experienced their stay at the ICU?

Regarding the follow-up, have you been missing anything?

Have you been in touch with any groups, organizations or support units that have been helpful for you?

Do you have any advice for others experiencing a similar situation?
