# Supplementary material for: The Caregiver Pathway, a Model for the Systematic and Individualized Follow-up of Family Caregivers at Intensive Care Units: Development Study
Source: JMIR Form Res. 2023 Apr 25;7:e46299. doi: 10.2196/46299 (PMC10170368; doi:10.2196/46299)
Supplement: Multimedia Appendix 2 [file formative_v7i1e46299_app2.pdf]

**ARTICLE**	**FINDINGS**	**ADDRESSED IN THE MODEL**
Blom, H., C. Gustavsson, and A.J. Sundler, *Participation and support in intensive care as experienced by close relatives of patients: a phenomenological study.* Intensive Crit Care Nurs, 2013. **29**(1): p. 1-8.	Highlighted the importance of support from the nurses and involvement in patient care.	Inspired the model development.<br>Question included in the assessment tool
Burton, L. C., Newsom, J. T., Schulz, R., Hirsch, C. H., & German, P. S. (1997). Preventive health behaviors among spousal caregivers. *Prev Med, 26*(2), 162-169.	Caregivers do not get enough rest, time to exercise or recuperate, and forget to take prescribed medications. A strong sense of control leads to better health outcomes among caregivers.	Question included in the assessment tool and in the manual for the follow up conversation. Text concerning self-care was included in the supportive card.
Choi, J., Donahoe, M. P., & Hoffman, L. A. (2016). Psychological and Physical Health in Family Caregivers of Intensive Care Unit Survivors: Current Knowledge and Future Research Strategies. *J Korean Acad Nurs, 46*(2), 159-167.	Caregivers are at risk for sleep disturbances, fatigue and poor self-care.	Inspired the model development.<br>Question included in the assessment tool and in the manual for the follow-up conversation. Text concerning self-care was included in the supportive card.
Czerwonka, A. I., Herridge, M. S., Chan, L., Chu, L. M., Matte, A., & Cameron, J. I. (2015). Changing support needs of survivors of complex critical illness and their family caregivers across the care continuum: a qualitative pilot study of Towards RECOVER. *J Crit Care, 30*(2), 242-249.	Caregivers need information, are worried, have anxiety and uncertainty. These needs vary and change through the stay at the intensive care unit (ICU) and afterwards.	The need for follow-up from admission to after the hospital stay.
Davidson, J. E., Aslakson, R. A., Long, A. C., Puntillo, K. A., Kross, E. K., Hart, J., . . . Curtis, J. R. (2017). Guidelines for Family-Centered Care in the Neonatal, Pediatric, and Adult ICU. *Crit Care Med, 45*(1), 103-128.	Guidelines for Family-Centered care.	Inspired the development of the entire model.
Engstrom A, S. S. (2004). The experiences of partners of critically ill persons in an intensive care unit. *Intensive Crit Care Nurs., 20*(5), 299-308.	To be present, respect for the patient, support from family and friends, to understand and accept what was happening, and to. receive information and hope were important factors for the caregivers.	Questions included in the assessment tool. Underlined the importance of the conversation with the ICU nurse after using the assessment tool.
Epstein, E. G., & Wolfe, K. (2016). A preliminary evaluation of trust and shared decision making among intensive care patients' family members. *Appl Nurs Res, 32*, 286-288.	Highlights the importance of trust and shared decision making.	Questions added in the assessment tool and followed up in the conversation with the registered nurse afterwards.
Johnson D, W. M., Cavanaugh B, et al. (1998). Measuring the ability to meet family needs in an ICU. *Crit Care Med., 26*(2), 266-271.	Having an assigned nurse was of importance	Encouragement to have the same registered nurse follow-up the caregivers.
Jones, C., Bäckman, C., & Griffiths, R. D. (2012). Intensive care diaries and relatives' symptoms of posttraumatic stress disorder after critical illness: a pilot study. *Am J Crit Care, 21*(3), 172-176.	The importance of writing a *Diary* for the patient, and the Diary could be useful for the follow-up of caregivers as well	Encouragement to write in diary for the patient as a supportive act for the caregivers as well, continues as existing practice.

Kentish-Barnes, N., Chevret, S., Champigneulle, B., Thirion, M., Souppart, V., Gilbert, M., . . . Azoulay, E. (2017). Effect of a condolence letter on grief symptoms among relatives of patients who died in the ICU: a randomized clinical trial. *Intensive Care Med, 43*(4), 473-484.	Condolence/sorry for your loss letter from the hospital did not have a positive effect, rather impacted the caregivers negatively.	The importance of personal follow-up and that an individual approach is needed to care for bereaved caregivers.
Matt, B., Schwarzkopf, D., Reinhart, K., König, C., & Hartog, C. S. (2017). Relatives' perception of stressors and psychological outcomes - Results from a survey study. *J Crit Care, 39*, 172-177.	It is important to identify caregivers who feel overburdened.	Inspired the model development.<br>Question added in the assessment tool and followed-up by the ICU nurse afterwards.
Serio, C. D., Kreutzer, J. S., & Witol, A. D. (1997). Family needs after traumatic brain injury: a factor analytic study of the Family Needs Questionnaire. *Brain Inj, 11*(1), 1-9.	Describes caregivers' needs addressed in the Family Needs Questionnaire:<br>Health information, Emotional support, Instrumental support, Professional support, Community Support network, Involvement with care.	The Family Needs questionnaire inspired the development of questions for the assessment tool.
Thompson, R., Boyle, D., Teel, C., Wambach, K., & Cramer, A. (1999). A qualitative analysis of family member needs and concerns in the population of patients with burns. *J Burn Care Rehabil, 20*(6), 487-496.	Timely questions to the caregivers: What are your major concerns? What is helping you the most? What kind of support do you need but are currently not receiving?	Inspired the question included in the assessment tool as well as the manual for the follow up conversation.
van Beusekom, I., Bakhshi-Raiez, F., de Keizer, N. F., Dongelmans, D. A., & van der Schaaf, M. (2016). Reported burden on informal caregivers of ICU survivors: a literature review. *Crit Care, 20*, 16.	Describes caregivers' burden at 6 months past an ICU discharge:<br>Depression, Anxiety, PTSD, Loss of employment, Financial burden, Lifestyle interference, Low health-related quality of life	Inspired information included about self-care and where to seek help in the supportive card.<br>Inspired the questions included in the manual for the follow-up conversation.
Wetzig K, M. M. (2017). The needs of families of ICU trauma patients: integrative review. *Int Crit Care Nurs.*(41), 63-70.	Identified the following themes as important for the caregivers:<br>Information, making sense, hope, support, involvement, protection	Inspired the question included in the assessment tool, the information included in the supportive card and questions included in the manual for the follow-up conversation.
Wilder Schaaf, K. P., Kreutzer, J. S., Danish, S. J., Pickett, T. C., Rybarczyk, B. D., & Nichols, M. G. (2013). Evaluating the needs of military and veterans' families in a polytrauma setting. *Rehabil Psychol, 58*(1), 106-110.	The need for Information,<br>The need for emotional support and instrumental support are often unmet.	Inspired question included in the assessment tool and the ensuing follow-up conversation with the nurse.
